# Evaluating factors that impact scoring an open response situational judgment test: a mixed methods approach

**DOI:** 10.3389/fmed.2024.1525156

**Published:** 2025-01-06

**Authors:** Muhammad Zafar Iqbal, Rodica Ivan, Colleen Robb, Jillian Derby

**Affiliations:** Research Department, Acuity Insights, Toronto, ON, Canada

**Keywords:** situational judgment tests, open response scoring, construct validity, professionalism, personal skills, professional skills, admissions

## Abstract

**Introduction:**

Situational judgment tests (SJT) are commonly used in admissions to measure skills associated with professionalism. Although open-response SJTs have shown strong psychometric properties, assessors’ personal beliefs, experiences, and cultural backgrounds may influence how they perceive, organize and evaluate information within test takers’ diverse responses. Additionally, SJT research typically focuses on reliability and predictive validity, whereas the construct validity of open response SJTs remains underexplored. This mixed methods study aims to address this gap by exploring the construct-(ir)relevant factors that may impact assessors’ evaluation of professionalism in open response SJTs.

**Methods:**

For this study, we used data from Casper, an open response SJT commonly used in professional program admissions. In Study I, a quantitative content analysis was conducted on 160 responses to identify factors which were significant predictors of low and high scores. Correlation coefficients and logistic regression models were used to evaluate the relationship between each factor and response scores. In Study II, think-aloud activities were conducted with 23 Casper assessors to directly observe how they evaluated responses. All interviews were transcribed verbatim, which were then thematically analyzed using an inductive coding technique.

**Results:**

Results from both the content analyses and think-aloud activities revealed that several construct relevant factors influenced scores. Scores were impacted by the extent to which test takers demonstrated the competencies probed for by the SJT, engaged with the context of the presented ethical dilemma, provided in-depth justifications for their response, considered various perspectives relevant to the presented dilemma, and provided creative solutions or insightful arguments for the suggested approach. Mixed results were found with respect to construct irrelevant factors, such as the flow, cohesion, and kinds of phrases used in the response.

**Conclusion:**

This mixed methods study contributes to the construct validity of SJTs by investigating construct relevant and irrelevant factors that may impact assessors’ evaluation of open responses. The findings of this study provide evidence that open-response SJTs are valid approaches to measure professional competencies more broadly, both in terms of *what* test takers focus on in their responses, as well as in terms of *how* they construct their responses.

## 1 Introduction

Situational judgment tests (SJT) are typically used to measure skills associated with professionalism by evaluating one’s responses to a variety of hypothetical scenarios that professionals would likely encounter in their roles or everyday life (1–3). Over the years, higher education programs have been increasingly integrating SJTs into their admissions process alongside traditional academic measures (e.g., GPA) across different professions, including medicine (4–7), healthcare (8, 9), and teacher’s education (10, 11). Historically, higher education programs relied on reference letters, personal statements, interviews, and/or multiple mini interviews (MMIs) to assess applicants’ personal and professional skills. Unfortunately, studies suggest that reference letters and personal statements have poor reliability and predictive validity (5, 12, 13). More recent concerns include the authenticity of such documents, given that letters written by artificial intelligence tools (i.e., ChatGPT) may sometimes be indistinguishable from those written by humans (14, 15). Alternatively, although interviews and MMIs are reliable and valid (16), they are also time and resource intensive for programs and applicants (9, 17). In the context of the limitations of these traditional measures, SJTs have emerged to be a cost effective and psychometrically sound method for assessing applicants’ professionalism early in the admissions process (5, 6).

While SJTs may share similar theoretical frameworks (2), there are unique test design elements that can have a notable impact on the measured construct(s) and on the quality of the test results (18, 19). We discuss two of these elements here: the response format and the question type. Typically, SJTs have either a fixed-response or an open-response format (and sometimes a combination of both). Fixed-response formats (e.g., multiple choice) require test takers to rate, rank, or select a response from a set of predetermined options, whereas open-response formats allow test takers to formulate their own responses by describing their approach and providing a rationale unique to them, their experience, and their interpretation of the presented situation (20, 21). Another key design difference in SJTs is the question type. Knowledge type questions, more commonly used in fixed-response SJTs, ask test takers to determine the extent to which the provided options would be effective in a specific scenario, inherently measuring one’s knowledge (22). Alternatively, behavioral tendency questions, more commonly used in open-response SJTs, ask test takers to express how they would likely react in response to the presented situation, thus measuring non-technical constructs like behaviors and traits associated with professionalism (22, 23).

When comparing the qualities of fixed-response and open-response SJTs, fixed-response formats have shown to produce larger demographic group differences (21); to be more vulnerable to fake or deceptive responses (15, 24, 25); and have revealed weaker predictive validity (26–28). In the context of medical school admissions, open-response SJTs measuring social intelligence and professionalism have evidenced correlations with interview performance ranging from *r* = 0.11 to *r* = 0.48 (27, 28), while fixed-response SJTs measuring similar constructs have evidenced relatively lower correlations ranging from *r* = 0.09 to *r* = 0.11 (26).

While open-response SJTs might be associated with relatively stronger psychometric properties, they are also inherently subject to more variability in the response content, and consequently more variability in how these responses are assessed. Open-response tests not only allow test takers to provide diverse and complex responses, they also offer more nuance in how assessors interpret and evaluate these individual responses against the provided scoring criteria (29–31). In a study investigating how assessors mark student essays, Hasan and Jones used think-aloud interviews to explore the scoring process and found that assessors would often rely on norm referencing (comparing essays to each other) even when instructed to closely follow scoring guidelines (31). Moreover, assessors’ personal beliefs, experiences, and cultural backgrounds may influence how they perceive, organize and evaluate information within responses and can sometimes lead to inconsistent or biased scoring (30, 32, 33). For instance, Condor found that construct-irrelevant factors such as response length, language, and phrase frequency can predict human assigned scores even for cognitive mathematics tests using open responses (34). Additionally, Mello et al. (35) argue that scoring open-ended responses is time-consuming, which might lead assessors to superficially screen responses and not fully consider applicants’ abilities. Other assessor related biases such as the halo effect (an applicant’s first impression influencing an assessor’s subsequent judgments) and leniency or severity bias (assessors consistently scoring higher or lower than average) have also been reported in the literature (33, 36). Given that this variability can undermine the reliability and fairness of SJT scores, it is vital that we understand the cognitive processes of assessors. It is important to highlight, however, that scoring can be quite complex for open-response and fixed-response SJTs alike. By design, SJTs present test takers with situations where several answers are plausible and/or appropriate. Since there is no definitive correct answer in either SJT format, fixed-response SJTs may employ several possible methods (i.e., empirical, expert-based, etc.) to score each response option, and thus impact the validity of the test (37), its reliability (38), as well as the measured construct (23).

Although both the response format (open/fixed) and the scoring process have been argued to affect the measured construct (19, 23), SJT research has primarily focused on (i) fixed-response SJTs, and (ii) quantitative psychometric properties, especially reliability and predictive validity evidence – that is – the relationship between SJT scores and future performance (6, 39). In fact, some scholars have claimed that the research focus on the relationship between SJT scores and other metrics (e.g., grades, interviews) has led to a lack of clarity in terms of the actual construct that SJTs are intended to measure (19, 40), which highlights the need for more in-depth construct validity research (19, 41). To address the literature gap on *how* SJTs measure their intended construct, Wolcott et al. (42) used think-aloud interviews to probe further into factors that impact test takers’ response process in a *fixed-response* SJT. In this study, we further address this gap by considering an entirely different piece of the puzzle, namely the factors that impact assessors’ scoring process in an *open-response* SJT. While SJTs often employ scoring guidelines to enhance score reliability and inter-rater agreement, assessors might nevertheless consider additional response attributes when deciding which scores to assign (30–32). Thus, we narrow in on the following research question: *which construct-(ir)relevant factors play a role in assessors’ evaluation of test takers’ unique answers to open-response SJT scenarios?*

To explore this research question, the Casper SJT was used, an open-response SJT that assesses the social intelligence and professionalism of those applying to a variety of professional programs (e.g., medicine, engineering, teacher’s education, health sciences, business, etc.). The Casper test comprises hypothetical scenarios designed to assess a combination of multiple personal and professional competencies including empathy, communication, motivation, resilience, self-awareness, problem-solving, collaboration, ethics, equity, and professionalism (43). These scenarios are presented to test-takers as either a text prompt (i.e., a short written description of a situation) or a video prompt (i.e., trained actors performing a situation). Test takers are required to respond to the scenario questions in two unique ways: either typing out their response or verbalizing their response via audio-visual recording. Responses are then scored by trained human assessors on a scale of 1 to 9. While assessors are provided with scoring guidelines specific for each scenario, scoring is also norm-referenced (i.e., responses are scored relative to other responses to the same scenario within the same test sitting). The scoring guidelines provide assessors with (1) a set of guiding questions to help them determine the extent to which the responses effectively answered the questions posed in the scenario and (2) detailed context on how the scenario relates to Casper competencies. Importantly, Casper was also selected because it demonstrates high reliability and validity (*α =* 0.82, test–retest reliability y = 0.75) (44), indicating that the items of the test work together to measure the same construct (45). Additionally, Casper has been shown to predict future performance on similar measures such as interview performance with correlations ranging from *r* = 0.11 to *r* = 0.48 (27, 28, 44).

Casper, with its open-response format and use of behavioral tendency questions, produces large pieces of text data which are then evaluated by human assessors. Although Casper has continuously demonstrated strong psychometric qualities (46), it is unclear what additional response attributes assessors consider during the scoring process (30, 31). By nature of its design, Casper provides optimal data for examining which construct-(ir)relevant factors may impact assessors’ evaluation of test takers’ unique answers to open-response SJT questions.

Given the complexity of this task, we employed a mixed-methods approach. First, we conducted a quantitative content analysis of test takers’ Casper responses to identify potential factors and response characteristics that might impact scoring (Study I). Then, in line with Hasan and Jones (31) and Wolcott et al. (42), we used *think-aloud interviews* to observe participants’ process and delve deeper into their decision making (Study II). While in Study I we focused on the content of test takers’ responses, in Study II we directly observed how assessors interacted with this content as they evaluated responses against scoring guidelines. Together, these findings shed more light on the implicit factors that play a role in assessing open-response SJTs, contribute to construct validity research on SJTs, and help enhance the transparency of SJTs more broadly.

## 2 Study I: quantitative content analysis

The primary aim of this study was to delve deeper into the content of test takers’ responses to Casper items and identify which response characteristics might play a role in scoring. Given the variability in response content and response assessment of open-response SJTs discussed above, we hypothesized that construct relevant and construct irrelevant factors alike impact a response’s score. Therefore, we expected that both types of factors would correlate with scores and predict low (1–3) and high (7–9) Casper scores.

### 2.1 Method

The open-response format of the Casper test results in large pieces of text data; therefore, we chose to conduct a content analysis because of its ability to dissect and identify granular response characteristics and apply a quantitative approach to aid in interpretation (47, 48). At its core, content analysis is a process by which large pieces of information are segmented into unique categories (i.e., factors) using coding rules which are guided by theory and/or previous findings (47, 49).

### 2.2 Procedure

We used a robust checklist for content analysis, which was developed by two researchers (RI and CR) who performed a thematic analysis of historical Casper data (i.e., 60 test takers responses) using an emergent approach. As outlined by Stemler (49), an emergent approach to checklist creation is an iterative process in which researchers review a set of data, identify factors for the checklist, reconcile differences, and edit accordingly. After developing the codes, we conducted multiple discussion rounds in which all four researchers went through all codes one by one, discussed their feasibility and voted for their inclusion and exclusion. In cases when a code was important but not clear, we reworded it to improve its clarity and applicability. Only those codes were kept in the checklist that received at least three out of four votes. Once the codes were finalized, two researchers (RI and CR) developed their definitions, and the other two researchers (MZI and JD) provided constructive feedback on the wording. Afterwards, consensus on the definitions of all codes was developed synchronously in a team discussion meeting.

Based on this historical data, we identified 13 different factors (see Table 1), nine of which were construct relevant and either related to information provided in the scoring guidelines (*Addressed competencies targeted in the scenario*, *Considered context of the scenari*o) or they were common construct relevant characteristics of responses which are not directly referenced in the scoring guidelines (provided justification, consideration of other perspectives). The construct irrelevant factors we identified in the responses pertained to linguistic considerations or to applicant appearance (for the video responses). One of these construct irrelevant factors, *Used phrases suggested by third party training materials,* was inspired by online unofficial sources (unaffiliated with Casper) which recommend using particular phrases, e.g., “I would approach my colleague in a non-confrontational manner in a private setting.”

**Table 1:** Checklist: emergent factors from the thematic analysis of historical data.

Theme	Factor	Factor levels
Construct relevant		
Demonstrated competencies	Addressed competencies targeted in the scenario	Failed to address competencies targeted in the scenario/Addressed some of the targeted competencies/Addressed all the targeted competencies
	Addressed additional competencies	Yes/No
Scenario engagement	Considered context of the scenario	Limited/Adequate/Excellent
	Insisted on lack of information	Yes/No
Justification and rationale	Vague rationale	Yes/No
	Depth of justification	No justification/Superficial/Limited/Clear & compelling
Perspective consideration	Considered perspectives	Considered one perspective/Briefly considered multiple perspectives/ Thoughtfully considered multiple perspectives
	Explicitly dismissed one side	Yes/No
Response quality	Provided insightful and/or unique arguments	Yes/No
Construct irrelevant		
Linguistic considerations	Noticeable grammatical errors (e.g., odd sentence structure)	Yes/No
	Used phrases suggested by 3rd party training materials (e.g., ‘non-judgmental manner’)	Yes/No
Video-response specific factors	Informal applicant appearance, clothing, and/or background	Yes/No
	Noticeable presence of longer pauses, silences, or disfluencies (“umm,” “err,” etc.)	Yes/No

### 2.3 Materials

To ensure a representative sample, the data selection process was as follows.

#### 2.3.1 Test selection

Casper tests were considered if they were from the most recent application cycle available (2022–2023) and written by a minimum of 1,000 test takers to a variety of health sciences programs (e.g., occupational therapy, physician assistant, nursing, etc.). Ultimately, we selected a test from June 2022 which featured responses from 1,264 unique US test takers.

#### 2.3.2 Scenario selection

The selected test included responses to 9 typed responses and 6 video responses. A sample of 3 scenarios of each response type were selected. We aimed for scenarios which had a good balance in terms of three psychometric criteria: average scores, item total correlations, and magnitude of demographic group differences.

The average score for the 15 scenarios ranged from 4.66 to 5.60; we selected scenarios that had an average score closer to the overall average score, namely 5.09. Aiming to ensure that a test taker’s score for a particular scenario was representative of their overall score, we only considered scenarios with an item total correlation between 0.30 and 0.70 as this range is often considered acceptable (50). All scenarios under consideration met this threshold, as their item total correlation ranged from 0.31 to 0.67. The magnitude of demographic group differences was assessed via Cohen’s *d* values. We aimed for the demographic differences for the score obtained on these particular scenarios to be considered negligible, small, or moderate in magnitude. For this reason, we ensured that no scenarios produced Cohen’s *d* values above 0.60 (51).

Following the identification of scenarios that met the desired quantitative thresholds, the final selections were discussed among the team to develop consensus. In total, six unique scenarios were selected for analysis: three typed responses and three video responses.

#### 2.3.3 Response selection

Using the checklist (see Table 1), three researchers (MZI, RI, CR) conducted the content analysis independently, resulting in 243 observations (81 responses x 3 researchers) for typed responses and 237 observations (79 responses x 3 researchers) for video responses (2 responses removed due to technological issues). To ensure that the responses used in the content analysis were representative of the responses typically observed within a Casper test, we considered both the response score and the response length. Within each response score category, responses were labeled as short, average, or long according to word count for typed responses or video length for video responses. That is, the first tertile by response length within each of the 9 score categories was labeled ‘short’ (mean word count 168.2), the second tertile ‘average’ (mean word count 202.2) and the third tertile ‘long’ (mean word count 241.3). After this classification, for each possible score (1–9), a short, average, and long response was randomly selected. This process was completed for each of the six unique scenarios. After selection, two participants’ video-response answers were removed due to technological issues. Thus, a total of 160 responses were analyzed: 81 responses for typed responses and 79 responses for video responses. Prior to conducting the content analysis on the selected responses, all three researchers completed a practice content analysis on nine typed responses and nine video responses. Upon completion of the practice content analysis, the researchers met to align on and further establish consensus on factor definitions and levels.

#### 2.3.4 Study participants

This demographic information was collected through an optional self-reported survey that test takers complete immediately after their test. Consent to use test takers’ responses for this study was obtained through the test’s *Terms and Conditions* which are signed upon test registration. This signed consent allows for response data to be used in research projects in an anonymized and aggregate fashion. In Table 2, we report the demographic makeup of the 154 unique test takers in the study sample, as well as the demographic makeup of the study population, namely the 31,860 applicants to US health science programs who took Casper in 2022–2023.

**Table 2:** Demographic makeup of participant sample.

	Study sample (*N* = 154)		Study population (*N* = 31,860)	
	*n*	%	*n*	%
Race				
Asian	26	21.31	3,515	14.73
Black, African, Caribbean, or African American	8	6.56	1,510	6.33
Hispanic, Latinx, or Spanish origin	14	11.48	2,949	12.36
Middle Eastern or Northern African	7	5.74	981	4.11
White or European	67	54.92	14,906	62.47
Another race, ethnicity, or origin/not answered	32	-	7,999	-
Gender				
Man	26	20.97	5,816	23.19
Woman	98	79.03	19,261	76.81
Other/prefer not to say/not answered	30	-	6,783	-
Age				
18–22	41	37.27	10,745	48.92
23–27	50	45.45	9,004	40.99
28 or older	19	17.27	2,216	10.09
Prefer not to say/not answered	44	-	9,895	-

Percentages in this table reflect only those who provided demographic information.

### 2.4 Data pre-processing

Prior to conducting the content analysis, responses were grouped into three score buckets: low (scores 1–3), average (scores 4–6), and high (scores 7–9). We then converted factor levels into numerical values. The majority of factors (69.23%, *n =* 9) were binary and were numerically coded as 1 for *yes* and 0 for *no*. Four remaining factors had more than two levels and were numerically coded according to the level definition. For factors in which a characteristic could be completely absent, the coding started with 0 and continued to increase as the characteristic became more apparent (see Table 3 for further detail).

**Table 3:** Multilevel factor coding.

Factor	Factor levels	Numerical code
Addressed competencies targeted in the scenario	Failed to address competencies targeted in the scenario	0
	Addressed some of the targeted competencies	1
	Addressed all the targeted competencies	2
Considered context of the scenario	Limited	1
	Adequate	2
	Excellent	3
Depth of justification	No justification	0
	Superficial	1
	Reasonable	2
	Clear & compelling	3
Considered perspectives	Considered one perspective	1
	Briefly considered multiple perspectives	2
	Thoughtfully considered multiple perspectives	3

Given that the three researchers evaluated the same 81 responses, the researcher evaluations were averaged for each factor for each response to avoid treating evaluations of the same responses as independent items. For example, if two researchers labeled a response as having a limited depth of justification (coded as 2) and one researcher labeled the same response as having a superficial depth of justification (coded as 1), the average score for the depth of justification for this particular response would be 1.67. The value of 1.67 was then used in the quantitative analyses as the factor score for that particular response.

### 2.5 Statistical data analysis

First, the data were evaluated via descriptive statistics and Spearman correlation analyses. Spearman correlation coefficients were used to evaluate the relationship between each factor and response scores (1–9), as they allow users to measure the relationship between ranked variables (45). While correlation coefficients provide insight into the direction and strength of a relationship between the factors and scores, they are unable to explain the effect the two variables have on one another. Thus, for each content analysis factor, a single predictor logistic regression model was fit to predict (1) high scores (scores of 7–9) and (2) low scores (scores of 1–3), allowing us to estimate the likelihood of receiving a high and low score with each factor. All data analyses were conducted using RStudio Version 2023.3.0.386 (52).

### 2.6 Results

The descriptive statistics for each response characteristic across the typed-response scenarios and video-response scenarios are reported in Table 4. These include the mean, median, and standard deviation of the average researcher evaluation for each factor across all unique responses.

**Table 4:** Descriptive statistics of response characteristics.

Themes	Factors	Typed responses					Video responses				
		Mean	Median	SD	Min	Max	Mean	Median	SD	Min	Max
Demonstrated competencies	Addressed competencies targeted in the scenario	1.36	1.67	0.76	0	2	1.46	1.67	0.61	0	2
	Addressed additional competencies	0.27	0	0.37	0	1	0.46	0.33	0.43	0	1
Scenario engagement	Considered context of the scenario	1.55	1.33	0.58	1	3	1.93	2	0.56	1	3
	Insisted on lack of information	0.13	0	0.31	0	1	0.06	0	0.21	0	1
Justification and rationale	Vague rationale	0.19	0	0.32	0	1	0.24	0	0.37	0	1
	Depth of justification	1.58	1.67	0.79	0	3	1.7	2	0.81	0	3
Perspective consideration	Considered perspectives	2.01	2	0.67	1	3	2.1	2	0.66	1	3
	Explicitly dismissed one side	0.11	0	0.29	0	1	0	0	0.04	0	0.33
Response quality	Provided insightful and/or unique arguments	0.17	0	0.31	0	1	0.22	0	0.34	0	1
Linguistic considerations	Noticeable grammatical errors (e.g., odd sentence structure)	0.13	0	0.28	0	1	0.01	0	0.05	0	0.33
	Used phrases suggested by 3rd party training materials	0.2	0	0.31	0	1	0.1	0	0.27	0	1
Video response specific factors	Informal applicant appearance, clothing, and/or background	NA	NA	NA	NA	NA	0.03	0	0.13	0	1
	Noticeable presence of longer pauses, silences, or disfluencies (“umm,” “err,” etc.)	NA	NA	NA	NA	NA	0.23	0	0.35	0	1

Below, we report the significant factors within each theme. Results from the correlation analyses are available in Table 5. Results from single-predictor bivariate logistic regression models that were fit are presented for each factor to assess the extent to which each could predict high scores and low scores are available in Tables 6, 7, respectively.

**Table 5:** Correlations between each factor and Casper scores.

Theme	Factor	Typed-response scenario		Video-response scenario	
		Correlation	*p*	Correlation	*p*
Demonstrated competencies	Addressed competencies targeted in the scenario	**0.59**	<0.001	**0.68**	<0.001
	Addressed additional competencies	**0.43**	<0.001	**0.38**	<0.001
Scenario engagement	Considered context of the scenario	**0.61**	<0.001	**0.60**	<0.001
	Insisted on lack of information	0.10	n.s.	−0.09	n.s.
Justification and rationale	Vague rationale	**−0.41**	<0.001	**−0.48**	<0.001
	Depth of justification	**0.62**	<0.001	**0.71**	<0.001
Perspective consideration	Considered perspectives	**0.51**	<0.001	**0.52**	<0.001
	Explicitly dismissed one side	**−0.34**	<0.001	−0.18	n.s.
Response quality	Provided insightful and/or unique arguments	**0.35**	<0.010	**0.47**	<0.001
Linguistic considerations	Noticeable grammatical errors (e.g., odd sentence structure)	−0.14	n.s.	−0.04	n.s.
	Used phrases suggested by 3rd party training materials	0.20	n.s.	−0.09	n.s.
Video response specific factors	Informal appearance, clothing, and/or background	NA	NA	0.03	n.s.
	Noticeable presence of longer pauses, silences, or disfluencies (“umm,” “err,” etc.)	NA	NA	−0.18	n.s.

Significant results (*p* < 0.05) bolded.

**Table 6:** Logistic regression models: predicting HIGH scores.

Theme	Factor	Typed-response scenarios				Video-response scenarios			
		Estimate	SE	*p*	OR	Estimate	SE	*p*	OR
Demonstrated competencies	Addressed competencies targeted in the scenario	1.77	0.55	0.001	5.88	4.07	1.12	<0.001	58.67
	Addressed additional competencies	1.88	0.65	0.004	6.57	2.17	0.63	0.001	8.77
Scenario engagement	Considered context of the scenario	2.49	0.57	<0.001	12.08	2.22	0.64	0.001	9.20
	Insisted on lack of information	0.13	0.77	0.863	1.14	−2.88	2.23	0.197	0.06
Justification & rationale	Vague rationale	−3.57	1.42	0.012	0.03	−2.98	1.11	0.007	0.05
	Depth of justification	1.52	0.40	<0.001	4.56	3.03	0.72	<0.001	20.70
Perspective consideration	Considered perspectives	1.60	0.44	<0.001	4.95	1.94	0.50	<0.001	6.94
	Explicitly dismissed one side	−3.74	2.33	0.109	0.02	−44.79^a^	4366.19^a^	0.992	NA
Response quality	Provided insightful and/or unique arguments	1.85	0.77	0.017	6.33	3.43	0.88	<0.001	30.97
Linguistic Considerations	Noticeable grammatical errors (e.g., odd sentence structure)	−0.92	0.99	0.350	0.40	−47.85^a^	5090.20^a^	0.993	NA
	Used phrases suggested by 3rd party training materials	1.49	0.76	0.049	4.42	0.21	0.87	0.814	1.23
Video response specific factors	Informal applicant appearance, clothing, and/or background	NA	NA	NA	NA	−2.20	2.86	0.441	0.11
	Noticeable presence of longer pauses, silences, or disfluencies	NA	NA	NA	NA	−2.08	0.95	0.028	0.12

^aVariables were observed in less than 5% of the analyzed data, likely resulting in extreme values.^

**Table 7:** Logistic regression models: predicting LOW scores.

Theme	Factor	Typed-response scenarios				Video-response scenarios			
		Estimate	SE	*p*	OR	Estimate	SE	*p*	OR
Demonstrated competencies	Addressed competencies targeted in the scenario	−1.28	0.35	<0.001	0.28	−2.50	0.57	<0.001	0.08
	Addressed additional competencies	−2.19	0.88	0.013	0.11	−1.30	0.61	0.034	0.27
Scenario engagement	Considered context of the scenario	−1.84	0.57	0.001	0.16	−2.53	0.63	<0.001	0.08
	Insisted on lack of information	−0.75	0.88	0.396	0.47	0.90	1.07	0.402	2.45
Justification & rationale	Vague rationale	2.28	0.80	0.004	9.79	2.76	0.73	<0.001	15.75
	Depth of justification	−1.73	0.45	<0.001	0.18	−1.91	0.47	<0.001	0.15
Perspective consideration	Considered perspectives	−1.32	0.42	0.002	0.27	−1.65	0.47	<0.001	0.19
	Explicitly dismissed one side	2.78	1.01	0.006	16.17	49.13	4366.19	0.991	NA
Response quality	Provided insightful and/or unique arguments	−1.38	0.94	0.143	0.25	−1.92	0.96	0.045	0.15
Linguistic considerations	Noticeable grammatical errors (e.g., odd sentence structure)	1.30	0.83	0.118	3.68	−47.50^a^	5090.20^a^	0.993	NA
	Used phrases suggested by 3rd party training materials	−1.65	0.97	0.087	0.19	0.37	0.87	0.672	1.45
Video response specific factors	Informal applicant appearance, clothing, and/or background	NA	NA	NA	NA	−47.85^a^	3705.94^a^	0.990	NA
	Noticeable presence of longer pauses, silences, or disfluencies	NA	NA	NA	NA	0.45	0.67	0.504	1.57

^aVariables were observed in less than 5% of the analyzed data, likely resulting in extreme values.^

#### 2.6.1 Demonstrated competencies

Responses that demonstrated the competencies of the test evidenced a positive and statistically significant relationship with scores. This is true for responses that addressed the competencies specifically targeted within the scenario (*r_Typed_ =* 0.59, *p < 0*.001; *r_Video_* = 0.68, *p < 0*.001), as well as for responses that addressed those which were not specifically targeted within that particular scenario (*r_Typed_ =* 0.38, *p < 0*.001; *r_Video_* = 0.43, *p < 0*.001). Further, demonstration of the competencies targeted in the scenario have greater odds of receiving a high score (OR*_Typed_* = 5.88, *p = 0*.001; OR*_Video_* = 58.67, *p < 0*.001). Responses which demonstrated other competencies that were not targeted in the scenarios also have greater odds of receiving a high score (OR*_Typed_* = 6.57, *p* = 0.004; OR*_Video_* = 8.77, *p* = 0.001).

#### 2.6.2 Scenario engagement

Results indicate that scores tended to increase as the level of consideration and integration of the scenario context increased (*r_Typed_ =* 0.60, *p* < 0.001; *r_Video_ =* 0.61, *p* < 0.001); responses that considered and integrated the scenario context were also more likely to attain a higher score (OR*_Typed_* = 2.22, *p* < 0.001; OR*_Video_* = 9.20, *p* = 0.001).

#### 2.6.3 Justification and rationale

Results indicate that providing a vague rationale had a negative impact on scores (*r_Typed_ =* −0.41, *p* < 0.001; *r_Video_ =* −0.48, *p* < 0.001) and increased the odds of receiving a low score (OR_Typed_ = 9.79, *p* = 0.004; OR_Video_ = 15.75, *p* < 0.001). Further, we found providing a higher-level justification had a positive impact on scores (*r_Typed_ =* 0.62, *p* < 0.001; *r_Video_* = 0.71, *p* < 0.001) and increased the odds of receiving a high score (OR_Typed_ = 4.56, *p* < 0.001; OR_Video_ = 20.70, *p* < 0.001).

#### 2.6.4 Perspective consideration

Scores are positively associated with the consideration of more perspectives (*r_Typed_* = 0.51, *p* < 0.001; r*_Video_* = 0.52, *p* < 0.001); responses that had a higher level of perspective consideration were more likely to achieve a high score (OR_Typed_ = 4.95, *p* < 0.001; OR_Video_ = 6.94, *p* < 0.001). Explicitly dismissing one or more of the perspectives in the scenario had a negative impact on scores for the typed responses (*r_Typed_ =* −0.34, *p* < 0.001) and increased the odds of receiving a low score (OR_Typed_ = 16.17, *p* = 0.006).

#### 2.6.5 Response quality

Scores are positively associated with responses which were found to provide an insightful and/or unique argument or approach to the presented dilemma (*r_Typed_ =* 0.35, *p* < 0.010; *r_Video_* = 0.47, *p* < 0.001) and these responses have greater odds of receiving a high score (OR*_Typed_* = 6.33, *p* = 0.017; OR*_Video_* = 30.97, *p* < 0.001). The presence of insightful and/or unique arguments or solutions evidenced lower odds of receiving a low score, although it was only significant for the video-response scenarios (OR*_Video_* = 0.15, *p* = 0.045).

#### 2.6.6 Linguistic considerations

Grammatical errors or the use of phrases suggested by 3rd party training materials were not significantly associated with scores. Also, the use of phrases suggested by 3rd party training materials was not predictive of low or high scores for video responses, however this factor achieved significance (*p* < 0.05) as a predictor of high scores for typed responses (OR*_Typed_* = 4.42, *p* = 0.049).

#### 2.6.7 Video-response specific factors

Informal applicant appearance and responses which contained noticeable pauses, silences, or disfluencies did not demonstrate a statistically significant relationship with scores. Only the presence of longer pauses, silences, or disfluencies (e.g., “umm,” “err”) was predictive of scores for video responses. Specifically, responses in which longer pauses, silences, or disfluencies were present had lower odds of receiving a high score (OR*_Video_* = 0.12, *p* = 0.028).

### 2.7 Discussion

The goal of this study was to analyze the content of test takers’ responses in order to identify which response characteristics evidenced a statistically significant relationship with scores. For the content analysis, we hypothesized that both construct relevant and construct irrelevant response characteristics would correlate with scores and that they would be significant predictors of low (1–3) and high (7–9) Casper scores.

The statistical analyses revealed that most of the construct-relevant factors identified in the checklist were indeed significant predictors of low and high Casper scores. The factors which related to the scoring guidelines (*Addressed competencies targeted in the scenario*, *Considered context of the scenario*) showed strong positive correlations with scores for both typed and video responses. These results provide evidence that assessors are mindful of the provided guidelines when scoring: responses which can demonstrate the targeted competencies (collaboration, empathy, etc.) in the scenario and which relate their response to the context of the scenario are associated with higher scores. In addition, we found that test takers who thoughtfully justified their approach to the presented situation, carefully considered multiple perspectives, and provided insightful and/or novel arguments or approaches in response to the presented situation were more likely to receive a high score. Opposite to this, test takers who provided a vague or neutral rationale for their approach to the presented situation were more likely to receive a low score.

With respect to the four construct irrelevant factors we identified in the checklist, correlation analyses did not reveal significant relationships with scores overall. On the other hand, logistic regression analyses revealed that *Use of phrases suggested by 3rd party training materials* was a significant predictor of high scoring typed responses, while *Noticeable presence of longer pauses, silences, or disfluencies* was a significant predictor of high scoring video responses (OR = 0.12) indicating *lower* odds of receiving a high score. Lastly, responses with *Noticeable grammatical errors* or *Informal applicant appearance, clothing and/or background* within the video responses did not have a relationship with or impact on scores.^1^

Given that the lack of statistical significance for some of these factors might also be due to low sample size, future research should replicate this study with larger sample sizes. In the interim, we conducted a post-hoc replication of this content analysis with a second Casper dataset to observe any differences due to geographical setting. Thus, we followed the same procedure laid out above and examined a different set of 81 typed responses from an Australian Casper test. This post-hoc replication revealed similar results to the ones reported above: the same construct relevant factors exhibited strong positive relationships with scores, while construct irrelevant factors were not significantly associated with scores.

The results of this study contribute to the construct validity evidence for the Casper SJT and to open response SJTs more broadly from the perspective of the content of test takers’ responses. While we found that factors related to the scoring guidelines used by assessors (demonstrating competencies, relating the response to the scenario context) were indeed significant predictors of scores, so were additional construct relevant factors, including the provided justification, the consideration of different perspectives, and the presence of what were considered to be ‘insightful’ or ‘unique’ arguments. We expected that these factors would also surface in Study II, where we examined the construct validity of Casper from the perspective of the scoring process.

## 3 Study II: think-aloud sessions

While in Study I we identified common characteristics of test takers’ responses and whether these characteristics demonstrated statistically significant relationships with scores, in Study II we approach construct validity from the perspective of the assessors and how responses are evaluated by directly observing the scoring process. Based on the findings of our content analysis, we hypothesize that, in addition to the scoring guidelines, assessors might factor in additional construct relevant considerations (e.g., provided justification). Although construct irrelevant factors did not reveal strong relationships with scores in Study I, we hypothesize that response characteristics which emerged in the content analysis (language, appearance, etc.) might also be noted by assessors and could play a role in scoring.

### 3.1 Method

To observe assessors’ scoring process and decision making, in line with similar research of this kind (31, 42), we used *think-aloud interviews*. *Think-aloud* is a method in which participants verbalize their thoughts, rationale and process while performing a given task (typically of higher-order thinking), or recall thoughts immediately following completion of that task (53). The theoretical underpinning of this method is that the thoughts elicited by the participants in real time are a valid reflection of the thoughts involved in the mediation of the task being performed (54). To capture assessor behaviors and to avoid cognitive overload, we split the think-aloud sessions into two phases: one think-aloud activity specifically targeted typed responses and another targeted video responses.

#### 3.1.1 Scenario and response selection

For the think-aloud activities, we selected scenarios and responses from the data that were used in the content analysis. The three researchers who conducted the content analysis identified lower, average, and higher scoring responses that were hypothesized to elicit rich discussion from the think-aloud participants.

#### 3.1.2 Participants

We recruited participants via a survey sent out to Casper assessors. Since the responses used in the think-aloud activities were from a North American Casper test, we recruited assessors from the US and Canada. The survey also included demographic questions (i.e., gender, race, age, geography, education level) to ensure that the selected participants were representative of the assessor population. 72 unique assessors expressed interest in participating in the think-aloud activity. When selecting participants, we used a stratified random sampling technique to ensure representation across demographic groups (i.e., race, gender, age) was approximately proportional with the total population of survey respondents. Ultimately, a total of 23 assessors participated in the two think-aloud sessions: 15 participated in the activity using typed responses, 4 in the activity using video responses, and 4 participated in both activities (see Table 8 for participant demographics). This resulted in 27 total think-aloud sessions.

**Table 8:** Demographic makeup of think-aloud participants.

	Study sample (*N* = 23)		Survey respondents (*N* = 72)	
	*n*	%	*n*	%
Race				
Asian	4	17.4	9	12.5
Black, African, Caribbean, or African American	5	21.7	8	11.1
White or European	12	52.2	41	56.9
Another race, ethnicity, or origin	2	8.7	14	19.4
Gender				
Man	3	13	12	16.7
Woman	19	82.6	57	79.2
Other/prefer not to say	1	4.7	3	4.2
Age				
25–34	5	21.7	24	33.3
35–44	6	26.1	17	23.6
45–54	6	26.1	14	19.4
55–64	2	8.7	8	11.1
65–74	3	13	7	9.7
Other/not answered	1	4.3	2	2.8

### 3.2 Procedure

Recruited assessors participated in the think-aloud study conducted from May to August, 2023. The Independent Review Board at Veritas IRB reviewed and approved this study (2023-3267-14464-1) on May 2, 2023. We obtained written informed consent for recording each session from all participants before the think-aloud activity, and participants also gave verbal consent during the activity. Each session lasted approximately 1 h, and all participants were compensated 50 USD or 60 CAD (depending on their country of residence) for their contribution to the study. In the first 10 min of each session, assessors were informed about the study objectives, the task expected of them, their role and the researcher’s role during the activity, and their rights as study participants.

During the think-aloud activity, participants first reviewed the scenario and read the associated scoring instructions and guidelines. They were then asked to review and score each of the four responses one by one and concurrently verbalize their thought process. To avoid leading the participants and compromising the integrity of the results, the researchers did not ask any questions or interfere during the think-aloud portion of the session. Researchers were only responsible for recording the assigned scores (record maintained separately) and noting down any extraordinary observations or comments made by the assessors during the think-aloud.

After completion of the think-aloud activity, researchers conducted a short exit interview to probe for any information that was of interest, but not already offered by the assessor. The exit interview questions were designed to explore general scoring behaviors and potential assessor biases. The complete think-aloud activity guide, including follow up questions and exit interview guide (for both typed and video responses) is given in the Supplementary material.

### 3.3 Data analysis

Prior to data analysis, all interviews were transcribed verbatim using Whisper, an AI-based, automatic speech recognition platform (55). These transcripts were proofread and cleaned by all three researchers individually to ensure accuracy. We did this by playing the recording while simultaneously reading through the transcript and correcting anything that was missing or transcribed incorrectly.

After cleaning the transcripts, we analyzed the data using an inductive coding technique (56). Each researcher coded the next researcher’s transcripts to ensure transparency and avoid researcher bias (57). These coded transcripts were then proofread by the other two researchers to ensure that we did not miss anything significant while coding the data.

After developing group consensus on coding, one researcher (MZI) grouped the codes into three categories (low, average and high scoring responses) based on the scores participants provided during the think-aloud activity and then developed themes and factors. Each theme represents the overarching domain and the factors represent the factors that underpin that domain. The other three researchers (RI, CR, JD) thoroughly reviewed the themes and factors and provided iterative feedback for improvement. The same data analysis approach was used for both think-aloud sessions (typed and video responses).

### 3.4 Results

Throughout the think-aloud sessions, seven themes were identified. The most prominent themes identified across all three score categories (low, average, high) in both typed and video response think-aloud sessions were: *demonstrated competencies, scenario engagement, justification and rationale, perspective consideration,* and *response quality*. *Linguistic considerations* was a common theme in both low and average scoring categories, whereas *concerning behaviors* was found in the low scoring category only. Table 9 provides all themes and factors alongside their associated frequencies. Below, each theme is described briefly.

**Table 9:** Themes and factors for all three scoring categories and associated frequencies (i.e., how often a factor was mentioned).

Themes and factors for LOW scores	Themes and factors for AVERAGE scores	Themes and factors for HIGH scores
Demonstrated Competencies Failed to address targeted competencies [11] Mentioned competencies (e.g., “empathy,” “ethical”) in responses but did not demonstrate them [4] Scenario Engagement Misinterpreted and/or showed limited understanding of the scenario/questions [15] Did not sufficiently engage with the scenario context/questions [3] Justification and Rationale Provided vague and/or unclear justification without specific/concrete examples [18] Provided generic explanation or solution [3] Perspective Consideration Failed to consider multiple perspectives [11] Imposed preconceived ideas or showed rigid thinking without acknowledging complexity of the situation [4] Explicitly dismissed others’ perspective(s) [2] Response Quality Unnecessarily repeated the scenario question [6] Lacked creative and/or insightful arguments [6] Provided repetitive statements without additional content [4] Provided limited/simplistic solutions [3] Linguistic Considerations Used phrases typically suggested by third party preparatory materials (i.e., “I would approach X in a calm and non-judgmental manner)” without backing with substantial content [10] Difficult to understand sentence/lacks coherence [3] Seemed rehearsed and robotic [1] Used condescending tone [1] Concerning Behaviors Used inappropriate language (i.e., uses derogatory terms, demonstrates misogyny, inequity and/or racism) [7] Showed lack of empathy [2]	Demonstrated Competencies Briefly addressed some of the targeted competencies [15] Scenario Engagement Demonstrated limited to reasonable understanding of the scenario context [7] Engaged with the scenario in a limited fashion [3] Misunderstood scenario context or question [2] Justification and Rationale Provided vague and/or unclear responses/explanation without specific/concrete examples [17] Provided limited or reasonable rationale/explanation, but not in depth [3] Perspective Consideration Focused on one perspective without acknowledging different viewpoints [4] Briefly considered multiple viewpoints or perspectives [4] Response Quality Provided a mix of strong and weak answers [19] Lacked creativity and/or originality [12] Provided creative, novel, or original ideas, arguments, or solutions [10] Unnecessarily repeated or paraphrased the scenario content [3] Sounded too rehearsed and robotic [2] Linguistic Considerations Used phrases typically suggested by third party preparatory materials (i.e., “I would approach X in a calm and non-judgmental manner)” without backing with substantial content [8]	Demonstrated Competencies Comprehensively demonstrated the targeted competencies [28] Demonstrated competencies above and beyond those targeted in the scenario [23] Scenario Engagement Showed clear understanding of and/or engagement with the scenario context [9] Fully understood and addressed the questions [5] Justification and Rationale Provided detailed, in-depth, reasoning/justification with nuance and complexity [23] Perspective Consideration Recognized and carefully considered multiple perspectives [16] Response Quality Provided insightful and/or practical solutions or arguments [19] Provided creative, novel or original ideas, observations or solutions [19] Provided multiple alternative solutions [11] Provided specific and clear strategies, and solutions [10] Provided diverse answers to scenario questions (not repeating the same points/content again) [3]

#### 3.4.1 Demonstrated competencies

Participants shared that the scores depended upon the extent to which test takers addressed competencies in their responses. For instance, high scoring responses comprehensively demonstrated the competencies targeted in the respective scenario and competencies beyond those that the scenario was probing for. Contrarily, the lower scoring responses failed to address some or all competencies. The two quotes below are two such instances of assessors explaining how demonstration of competencies impacted the score.

*“[The responses] were in tune with what we were expecting of them in terms of, you know, collaboration [and empathy], and they also showed things like the other competencies that were not necessarily [targeted in the scenario] like self-awareness or problem solving. […] They were good responses in my opinion.”* – (UR2; score 7).

*“there wasn’t a lot of problem solving [targeted aspect] in this situation for this first response. And so that’s going to make me tend to score lower because I know that’s one thing that we are looking for. Like, again, the scenario is intended to probe for problem solving and also resilience. So we are not really seeing a lot of that from this first response.”* (AR3; score 3).

#### 3.4.2 Scenario engagement

This theme represents the extent to which test takers incorporated the context of the scenario into their response and addressed the associated questions. Participants shared that high scoring responses showed clear understanding of the scenario; engaged well with the scenario context; and fully understood and addressed the questions. Contrarily, low scoring responses either misinterpreted or showed limited understanding of the scenario and/or the question(s); and did not engage sufficiently with the scenario context. One participant shared,

*“[High-scoring responses demonstrate] identification of complexity or nuance, where people aren’t giving very simplistic answers […] [and that] can be understanding the impact of context, right? […] something that shows that it’s beyond, you know, a reaction, that there’s some reflection”* (CR16; on high scores).

#### 3.4.3 Justification and rationale

This theme represents the depth of the justification for the provided approach in the response. Participants shared that high scoring responses provided detailed and in-depth reasoning and justification, took a stance on moral issues, and gave explanations for their position. Contrarily, low scoring responses provided vague and/or unclear justifications without specific or concrete examples. One participant said,

*“I feel like they could have gone into further detail. I would say a better response would have gone into more detail on how they can collaborate as a team […]. Just further explaining their response, you know. […]. Give examples.”* (UR17; score 4).

#### 3.4.4 Perspective consideration

This theme represents how test takers considered the perspectives of different parties within a scenario. Participants shared that high scoring responses typically recognized and considered multiple perspectives, whereas low scoring responses imposed preconceived ideas or showed rigid thinking without acknowledging the complexity of the situation; and explicitly dismissed others’ perspective(s). One participant shared,

*“[Low scoring responses are] too simple, um too polarized. […] Being dismissive of another perspective or another view or one of the people involved in the scenario. Um, just really uh, not giving any consideration to them or writing them off which I have seen, […] dismissing one of the players in this scenario too quickly.”* (AR5; on low scores).

#### 3.4.5 Response quality

This theme represents the quality of the provided argumentation or solution in the responses. Participants explained that high scoring responses provided creative, novel or original ideas, observations, or solutions to the presented dilemma; provided insightful and/or practical arguments or solutions; provided specific and clear strategies, or solutions; provided multiple alternative solutions instead of a single surface level solution; and provided diverse answers to the scenario questions instead of repeating the same points. Contrarily, low scoring responses provided limited (or simplistic) solutions; unnecessarily repeated the scenario information; provided repetitive statements without additional content; and lacked creative and/or insightful arguments. One participant said,

*“Also, when the person comes up with a different solution than what everybody else is saying, then I score higher. I feel like sometimes the easiest solution is the same thing everybody says. It’s the first thing that comes to mind. But when I hear somebody say something that’s something different and it makes me think like, wow, they are not really thinking about the usual stuff.” (UR14; on high scores).*

*“See, these are the kind of answers that I look for! Because I think you cannot always handle problems in, like, the same way. […] sometimes it kind of requires being unique and thinking outside the box.” (UR13; score 8).*

#### 3.4.6 Linguistic considerations

This theme subsumes language related aspects including sentence structure, coherence, clarity and conciseness. In particular, participants shared that low scoring responses were difficult to understand; lacked coherence; used phrases typically suggested by third party preparatory materials (i.e., “I would approach X in a calm and non-judgmental manner”) without supporting these statements with further content or rationale; used a condescending tone; and sounded rehearsed or robotic. The use of phrases from third party preparatory materials was also noted for average scoring responses. This theme did not emerge in the high scoring response category. One participant shared,

*“And in [the response] can occasionally lie the “canned” formula answers without anything additional provided, right? I’ve been to the prep course, I’ve learned that I’m supposed to say, I’m going to “approach you in a non-confrontational manner,” and then there’s nothing else.” (CR16; on low scores).*

#### 3.4.7 Concerning behaviors

This theme refers to test takers’ comments and/or behaviors that assessors found concerning, (e.g., unprofessional behavior, lack of empathy). Participants noted that low scoring responses can sometimes feature inappropriate language (i.e., used derogatory terms, demonstrated misogyny, inequity and/or racism) or insensitivity toward the individuals in the scenario. This theme only emerged in the case of low scoring responses.

### 3.5 Discussion

In Study II, we aimed to observe the scoring process while assessors evaluated typed and video responses in order to identify which factors pertaining to the responses might impact response scores. The results of this qualitative study with assessors largely corroborated the findings of our quantitative content analysis.

The most frequent theme that emerged during the *think-aloud* sessions and follow-up interviews was *Demonstrated Competencies.* As expected, based on the scoring guidelines, low scores were associated with perceived insufficient demonstration of these competencies, while responses that clearly demonstrated competencies were associated with high scores. Another construct relevant theme that was related to the scoring guidelines was *Scenario Engagement*. We observed that assessors tend to assign low scores to responses that show limited understanding of the presented dilemma, while high scoring responses engage with the scenario in more depth, and demonstrate complex and nuanced understanding of the dilemma.

Assessors also highlighted construct relevant response characteristics that were not included in the scoring guidelines directly; these characteristics correspond to the following themes: *Justification and Rationale, Perspective Consideration, Response Quality*, and *Concerning Behaviors*. Low scoring responses were frequently found to be vague, to be less considerate of the different perspectives in the scenario, to be repetitive, or to sometimes demonstrate lack of empathy or equity. On the other hand, high scoring responses were described as providing detailed justifications for their approaches, acknowledging and discussing different relevant perspectives to the presented dilemma, and as providing insightful, creative or multiple and diverse arguments and/or solutions.

Lastly, assessors flagged several construct irrelevant factors which we have listed under *Linguistic Considerations*. Some raters highlighted that low scoring responses may sometimes be unclear or lack coherence, or that they may sound “rehearsed” or seem “condescending.” The most commonly mentioned factor in this theme, however, had to do with the use of phrases suggested by 3rd party training materials. The qualitative interviews revealed that assessors are largely indifferent to test takers’ use of phrases such as “I would approach X in a calm and non-judgmental manner”; assessors were likely to find the use of these phrases generic and to assign responses including such phrases either low or average scores (see CR11 below).

*“Well, there’s a lot of [test takers who] are using, “I will talk to this person in a nonjudgmental, non-confrontational…” And I think those people have been coached to use that language. So, it’s not really annoying, but it’s not original and unique.”* (CR11).

Overall, the results of this second study contribute to the construct validity evidence for the Casper SJT and to open response SJTs more broadly from the perspective of response characteristics noted by assessors during scoring. Our results show that assessors were indeed mindful of the information provided in the scoring guidelines and that the demonstration of competencies and understanding the complexities of the presented scenario were important considerations during scoring. Additional construct relevant factors mentioned by the assessors included the clarity and/or insightfulness of the provided arguments and solutions, and how the test takers consider and incorporate others’ perspectives in their response. Lastly, we found that construct irrelevant factors like the use of specific phrases and the coherence of the response may also be considered during the scoring process.

## 4 General discussion

In the context of increased SJT use and research focused predominantly on SJT reliability, experts have highlighted a need for in-depth construct validity research (19, 40, 41). Typically, construct validity is estimated by examining the relationship between SJT scores and scores on assessments that measure similar constructs (3, 26). The counterargument to this approach is that the focus on what SJT scores relate to has led to a lack of clarity in terms of what SJTs actually measure (19, 40), and research to address this gap has been scant. To examine how SJTs measure their intended constructs, Wolcott et al. (42) explored test takers’ cognitive process as they responded to fixed-response SJT items. In this paper, we focused on a different and novel piece of the construct validity puzzle, namely how the construct relevant and irrelevant characteristics of test takers’ answers to open-response SJT items impact assessors and their scoring process. To address this gap, we used data from a commonly used open-response SJT in higher education admissions, Casper, which has strong psychometric properties (28, 46, 58).

Unlike fixed-response SJTs, where a test taker selects or ranks given responses, open-response SJTs allow test takers to freely respond to the question and provide reasoning and detail to their approach. This means that while open-response SJTs provide richer and more complex data, they also allow SJT assessors more room for interpretation when evaluating responses against the scoring criteria (30, 31), bearing similarity to the assessment of short essays (29). Given the complexity of these responses, we employed a mixed-methods approach. In Study I, we analyzed the content of archival Casper responses to identify potential factors and assess their statistical relationship with scores assigned by assessors. In Study II, we used *think-aloud interviews* to directly examine the scoring process, and how assessors interacted with the responses.

Results from both studies revealed construct relevant factors that relate to scores that may consciously or unconsciously impact scoring. First, both the quantitative content analysis on archival responses and the think-aloud activities with assessors reflected the instructions provided to assessors in the scenario-specific scoring guidelines. Namely, we found that test takers who demonstrated the competencies within the test construct (ethics, collaboration, etc.) and who demonstrated engagement and reflection on the context of the provided ethical dilemma were likely to obtain higher scores. Secondly, both studies revealed additional construct relevant factors, which are not directly related to the scoring guidelines. Results showed that assessors were mindful of the justification provided by test takers for their approach: responses with in-depth rationale were likely to receive higher scores, while vague responses were likely to receive lower scores. Analyzing the provided situation and providing a clear rationale is related to critical thinking (59, 60), which is an indirect component of two competencies targeted by Casper: problem solving and collaboration. Additionally, although not made explicit in the scoring guidelines, assessors also noted whether responses gave thoughtful or insufficient consideration to the various perspectives in the scenario, and whether they provided novel, insightful, creative, or generic and simplistic arguments and solutions. While perspective consideration is a facet of the Casper competency of empathy (61), the factor pertaining to creative or insightful or diverse arguments and solutions is again a reflection of problem solving and collaboration (60), as well as of critical thinking more broadly (62).^2^

While the correlations between scores and the four construct irrelevant factors identified during the content analysis did not achieve significance, logistic regressions in the content analysis revealed that video responses with noticeable pauses, silences, or disfluencies had lower odds of receiving a high score. Although such pauses were not mentioned by assessors during the think-aloud activities, a few assessors did note that responses that lack coherence or those which seem ‘rehearsed’ or ‘robotic’ are likely to receive low scores.

One assessor also noted that responses which have a ‘condescending tone’ may receive lower scores. While flow and cohesion may be construct-relevant in language assessments (29), they are not in the case of SJTs that measure social intelligence and professionalism. These findings bear similarity to those of Condor (34), who found that construct-irrelevant linguistic features (grammar, phrases) may impact the scores of open-ended responses on a mathematics assessment. In the case of our study, while the presence of grammatical errors did not have a significant effect on scores in either response format, linguistic aspects like tone, flow, and cohesion were salient.

Lastly, logistic regression analyses also showed that typed responses which included phrases suggested by third party training materials had higher odds of receiving a high score. On the other hand, the think-aloud activities and interviews with assessors revealed that the use of such phrases (e.g., “non-confrontational manner”) was found ‘unoriginal’ and assessors believed that these phrases were used as an effect of reviewing unofficial test prep materials. These ‘canned’ phrases used without supporting evidence or rationale often resulted in low or average scores. The mixed results might suggest that while these phrases are found in both low and high scoring responses, and while Casper assessors are not likely to reward the use of these phrases because they seem ‘canned’ or ‘empty’, test takers who receive high scores tend to provide compelling responses *despite* the use of these phrases. Our previous work showed that applicants who used official Casper sources to prepare for the test scored highest, applicants who used 3rd party training materials performed worse, and those who did not prepare at all performed the worst (44). Given this context and our findings, it is possible that the use of phrases like “non-confrontational manner” may not have a positive effect on scores, but may be correlated with whether test takers prepared or not for the Casper test. Future research can provide more insight into how various third-party preparatory methods may impact scores.

### 4.1 Limitations and future research

This study is, to our knowledge, the first of its kind to investigate the construct validity of open response SJTs by examining (i) which response characteristics are associated with scores, and (ii) which implicit factors influence scores during evaluation. Given that validity research typically focuses on fixed response SJTs and on psychometric analyses and external metrics and assessments that relate to SJT scores (6, 19, 39) or on test item instructions (63), it is difficult to draw parallels between the nature of our novel results and previous findings. While Wolcott et al. (42) broke new ground by examining the cognitive processes of test takers in the context of fixed response SJT, we examined the scoring process of assessors evaluating an open response SJT. We hope that future research will delve even deeper into the construct validity of these assessments by undertaking mixed-methods and qualitative studies on the process of test takers and assessors alike.

While we found evidence to support that performance on an open response SJT like Casper can be impacted by both construct relevant and construct irrelevant factors, these results have only been based on Casper data, and have not been investigated in the case of other open response SJTs. We hope that this paper provides a methodology that researchers may apply to other open response SJTs in the future.

Although the think-aloud method is highly beneficial in collecting data on assessors’ scoring process during their live evaluation of responses, this kind of study is subject to self-selection bias. It is possible that assessors who chose to participate in the study are more comfortable with the scoring guidelines, which is perhaps one reason why construct relevant factors related to the guidelines were featured so frequently. Additionally, it could be argued that this method, where an assessor is being observed while scoring, might preclude them from mentioning construct irrelevant factors. For instance, Casper assessors receive implicit bias training where they are instructed to not penalize grammatical errors, which might have prevented them from mentioning grammar during the think-aloud activities. Moreover, the participants of this study are regular assessors of the Casper test, and their role in test scoring could present a possible source of social desirability bias (where participants feel compelled to provide favorable answers rather than sharing their true opinion).

Another limitation of our research is the sample size. The content analysis was conducted on 160 responses and the think-aloud activity was conducted with 23 participants. It is possible that some factors are infrequent and might have achieved significance in a larger sample size. While the findings of the content analysis were corroborated in a post-hoc replication on a different set of 81 responses, we leave a larger scale replication of this study to future research.

### 4.2 Conclusion and implications

This mixed methods study contributes to the validity research of SJTs by investigating construct relevant and irrelevant factors that may impact assessors evaluating open response SJTs. Results from the quantitative content analyses of archival data and think-aloud activities with assessors reveal that several construct relevant factors have an effect on scores: both those which reflect the scoring guidelines, as well as additional implicit factors. We found that scores are dependent on the extent to which responses demonstrated the personal and professional competencies probed for by the SJT, engaged with the context of the presented ethical dilemma, provided in-depth justifications for their response, considered the various perspectives relevant to the scenario, and provided creative solutions or insightful arguments for their approach. We also found mixed results with respect to construct irrelevant factors, such as the flow, cohesion, and kinds of phrases used in the response.

This is the first study of its kind to analyze how response characteristics relate to construct relevant and irrelevant factors in open-response SJTs more broadly, but also for the specific test (Casper) that provided the data. With respect to Casper, we found that the two kinds of response formats (typed and video) were impacted by the same factors: the results were largely the same across the two formats. With respect to open-response SJTs, our study provides an approach for how to investigate construct validity by examining the content of the responses and the scoring processes of the assessors. Our results also provide evidence that open-response SJTs are valid approaches to measure professionalism related competencies such as empathy, collaboration, problem solving, and critical thinking more broadly both in terms of *what* test takers focus on in their response, as well as in terms of *how* they construct their response.

## Data Availability

Research data are not publicly available due to the confidentiality agreements with Casper applicants. However, the raw, anonymized data and analyses performed in the study are available from the corresponding author upon reasonable request.

## Footnotes

^1^It is important to note, however, that for video responses the factors *Noticeable grammatical errors* and *Informal applicant appearance, clothing, and/or background* were observed in less than 5% of the analyzed data. It is possible that these factors might achieve significance in a larger sample.

^2^These construct relevant factors were found in the case of both typed and video responses. The only difference between the two formats was found for the *‘Explicitly dismissed one side’* factor, which achieved significance in the content analysis of typed responses, but not in the content analysis of video responses. It is possible that either it is less likely for test takers to appear dismissive of other viewpoints in video responses, or that this characteristic was infrequent in the sample we analyzed.
