# Supplementary material for: Evaluating factors that impact scoring an open response situational judgment test: a mixed methods approach
Source: Front Med (Lausanne). 2025 Jan 6;11:1525156. doi: 10.3389/fmed.2024.1525156 (PMC11743161; doi:10.3389/fmed.2024.1525156)
Supplement: Supplementary file 1 [file Data_Sheet_1.docx]

# **Evaluating factors that impact scoring an open response situational judgment test: a mixed methods approach**

# **Supplemental Material: Think-Aloud Instructions & Interview Guide**

## **Introduction & Instructions**

Hello [name], welcome! How are you doing today?

My name is [name] and I am a researcher on this project. Thank you for accepting our request to participate in this research project! Also thanks for joining me today for the think-aloud exercise which will be followed by an exit interview!

Please let me give you some context of the project. Our project aims to explore the factors that play a role in scoring Casper responses. We want to reassure you that your identity will not be disclosed at any point of the study.

Is the study objective clear to you? *[if questions, answer]*

We're really grateful you've chosen to participate in this study! As we promised, you'll be receiving an Amazon gift card of 50 US/60 Canadian dollars once you complete the session. We will be in touch with you shortly after this session to share the gift card via email.

There are no known risks involved with your participation in this study. If you feel uncomfortable at any time, then you are free to opt out. But, if you choose to not complete the session, then you will not be compensated. Please remember that your choice to complete or not complete this exercise will not impact your role as an assessor at Acuity Insights.

Do you have any questions related to the study that I can answer for you before explaining the task?

I know you already consented in the informed consent form, but I would like to ask before we start: do you consent to record this session? *[Yes? start recording]*

Can you hear me and see my screen properly? *[Share mock responses and scoring guidelines. Yes? Move on; No? Make adjustments]*

Let’s also check that the audio sharing works. Can you hear this? *[Play a demo video for 5-10 seconds max. If everything is clear then move on; No? Make adjustments]*

The first part of today’s activity is a think-aloud exercise. We would like you to evaluate test taker responses, exactly as you typically do. However, the key difference is that we will ask you to speak your thoughts out loud as you reason out what score to assign. More specifically, what characteristics or elements of the response did you consider while scoring? What factors made you choose the score for the given response?

We are going to use one scenario today. You will assess 4 different test takers for this scenario. Then, after scoring the responses of these four test takers, you will be asked some follow-up questions based on this activity and your general experience.

Do you have any questions about this task so far?  *[Yes? Explain more; No? Move on]*

Today we really want to focus on your own thought process as you consider these specific responses, which is why we are only going to go over these four test takers. However, if you would like to go back and change a score at any point, just let me know! So far so good? Any questions?

After watching the scenario, I will show you both the scoring guidelines and the chosen responses side by side on my screen. If you ever need to see a different section of the document, please just let me know and I will scroll up or down. I can also replay sections of the video response whenever you need to.

So, in a nutshell, please share your thoughts about each response first and then assign a score to the response. This will help us understand what specific factors or characteristics played a role in the score you gave. What was good, what was not so good, what caught your attention?

There are no wrong or right answers or reasons, we really just want to find out about each assessor’s personal process. This is a safe space where you can openly share your thoughts and feelings. Once again, whatever you share will have no impact on your job as an assessor with Acuity Insights. So please, whatever thought comes to mind as you are considering which score to assign, let me know!

You will repeat the same exercise for each test taker response. I will not interfere while you are thinking aloud, so please feel free to voice your thoughts and let me know when to scroll up or down, or replay, but I will not be able to respond to questions until the end of the exercise.

Once again, the scoring guidelines will be on the right hand side *[Use mouse to show doc on the screen]* of the screen and the responses on the left *[Use mouse to show doc on the screen]*. Let me know if you ever want me to scroll *[Pretend scroll on the right]* or replay any parts of the video. Please remember to voice all of your thoughts as you evaluate the responses.

*[Screen share and show scoring guidelines and responses]*

We have scheduled about 5 to 10 minutes to score and think aloud for each test taker review, but it is no problem if you need more or less time. Is this task clear to you? Do you have any questions? *[Explain more if participant asks]*

Let’s start with the scenario. Here you go! First, is the scenario guidelines text clear and readable to you or do you want me to zoom in? *[zoom in if participant asks]* Great. I will now play the scenario video – please watch it carefully. *[Video starts on participant’s screen]*

Would you like me to play it again? *[Play again if participant asks]*

I’ll mostly try to remain silent as we go through the test taker responses so that you may voice your thoughts as you analyze the responses without me interrupting your thought process.

Are you ready to review the first set?

Great! I’ll get us started with the response to Question 1 for your review. Just let me know when you want me to click and play the response to Question 2. *[Play Response 1]*

Would you like me to play this first response again? *[Play again if participant asks]*

*[After participant says to move on to Response 2, play Response 2]*

*[If participant says nothing or pauses after a while:]*

Would you like to continue to Test Takers A’s 2nd response? *[Play Test Taker A Response 2]*

*[Optional questions IF participant DOES NOT think aloud when evaluating Test Taker A:*

*Are there any specific features of the response that stand out to you?*

*Could you say a bit about the reasoning for your scoring?]*

Shall we continue to the next responses? *[check if participant has any questions]*

*[Repeat process for Test Takers B, C, and D]*

Would you like to revise any of the scores you have already assigned?

## **Think-Aloud Follow-Up Questions**

*[After confirming scoring is complete for all responses, proceed with follow-up questions]*

I have a couple of questions related to the exercise before we move on to the interview.

1. Optional question: Could you say a bit about the reasoning for your scoring for this set of responses?
2. While rating response X, you mentioned Y. Could you speak more to that?
   1. How did it play a role in your scoring process?
3. When comparing the responses of Test Taker X and Test Taker Y, what were some key differences for you?

*[stop screen sharing once participant is done talking]*

Well, thank you very much for being so open and honest! That is the end of think-aloud exercise. Now we will move on to the interview. Would you like to take a short break? *[If participant asks for a break, break for 2-3 minutes, if not then continue]*

I will now ask you a few questions regarding your typical scoring process. Please be as open as you can so that we can learn from your experience. We are really looking for your honest thoughts and feelings and everything you say is completely valid and will not impact your role at Acuity Insights. So no judgments here! If any question is not clear to you then please ask and I will clarify it to the best of my ability. Shall we start?

Okay, here we go!

## **Exit Interview Guide**

1. What factors or characteristics do you feel typically contribute to a high scoring **typed/video** response for you?

[use ‘typed’ in think-aloud sessions with typed responses, ‘video’ for video responses]

- 1. Could you say more about X?
  2. Can you think of any specific examples?

1. On the opposite side of the spectrum, what factors or characteristics do you feel typically contribute to a low scoring **typed/video** response for you?

[use ‘typed’ in think-aloud sessions with typed responses, ‘video’ for video responses]

- 1. Could you say more about X?
  2. Can you think of any specific examples?

1. When scoring video responses in particular, is there anything you tend to notice outside of the content of the response? *(For example the applicant’s body language or appearance, appearance of the room, or the speaking skills of the applicant.)*

Potential Follow-ups:

1. How do you find X plays a role in your evaluations?
2. Can you think of any specific examples?
3. Let's talk a little bit about assessing typed responses vs. video responses. What are the most prominent differences between assessing typed versus video responses that play a role in your scoring?

1. Is there something you would like to add that I might have missed in my questions?

## **Thank You & Payment**

Thank you so much for your detailed answers! Do you have any questions before I end today’s call? Is the email address we used to schedule this appointment the right one to use for the gift card as well?

Thank you!

**Payment Note**

Somebody will reach out to you within the next two weeks. If you do not receive the gift card, please email us and we will put you in touch.
